# Does learning burnout link physical exercise and problematic short-video use? Evidence from a three-wave random-intercept cross-lagged panel model and supplementary machine-learning analysis among Chinese high school students

**DOI:** 10.3389/fpsyg.2026.1929181

**Published:** 2026-10-01

**Authors:** Tian Yin, Yanfeng Peng, Longjiang Chen, Zetian Wang, Xu Liu, Changcheng Xie, Zeng Gao

**Affiliations:** 1 Xiangtan University, Xiangtan, Hunan, China; 2 School of Physical Education, Yunnan Minzu University, Kunming, Yunnan, China; 3 School of Physical Education, Yunnan Normal University, Kunming, Yunnan, China; 4 Hezhou University, Hezhou, Guangxi, China

**Keywords:** high school students, learning burnout, machine learning, physical exercise, problematic short-video use, random-intercept cross-lagged panel model

## Abstract

**Background:**

Problematic short-video use has become a growing behavioral health concern among adolescents, yet its longitudinal links with physical exercise and learning burnout remain insufficiently understood. This study examined the within-person bidirectional associations among physical exercise, learning burnout, and problematic short-video use, tested whether learning burnout served as a longitudinal pathway linking physical exercise to later problematic short-video use, and explored the predictive value of these variables for subsequent problematic short-video use.

**Methods:**

A three-wave longitudinal survey with 6-month intervals was conducted among 1,327 students aged 16–18 years from five high schools in Yunnan Province, China. Physical exercise, learning burnout, and problematic short-video use were assessed using the Physical Activity Rating Scale-3 (PARS-3), the Adolescent Student Burnout Inventory, and the Short-Video Addiction Scale. Random-intercept cross-lagged panel modeling was used to separate stable between-person differences from within-person fluctuations while controlling for demographic and educational covariates. The longitudinal indirect association was estimated using 5,000 bias-corrected bootstrap samples. Supplementary ridge regression and extreme gradient boosting models, using nested cross-validation and a holdout test set, examined the incremental predictive value of physical exercise and learning burnout for later problematic short-video use.

**Results:**

At the between-person level, higher long-term physical exercise was associated with lower learning burnout and problematic short-video use, whereas higher learning burnout was associated with higher problematic short-video use. At the within-person level, higher-than-usual physical exercise predicted lower subsequent learning burnout and problematic short-video use. Learning burnout and problematic short-video use showed positive reciprocal associations over time, and both predicted lower subsequent physical exercise. The longitudinal indirect association from physical exercise to later problematic short-video use through learning burnout was statistically significant but small. In supplementary machine-learning analyses, the best holdout *R*^2^ was 0.311, but adding physical exercise and learning burnout did not improve prediction beyond prior problematic short-video use. SHAP results from the extended XGBoost model were therefore interpreted as exploratory feature attributions.

**Conclusion:**

Physical exercise, learning burnout, and problematic short-video use showed interconnected longitudinal associations among Chinese high school students. Learning burnout may represent one small longitudinal indirect pathway linking physical exercise to later problematic short-video use, while recent problematic short-video use provides the most informative signal for future risk. These findings may inform the development and evaluation of school-based health-promotion strategies that support physical exercise, address learning burnout, and respond to recent problematic-use symptoms.

## 1 Introduction

### 1.1 Problematic short-video use as an adolescent public health concern

In the digital era, short-video use has become deeply embedded in everyday life, reshaping how individuals obtain information, regulate emotions, engage in social interaction, and structure leisure time (Qi, 2024). Compared with traditional media, short-video platforms are particularly effective in capturing and sustaining users’ attention through algorithmic recommendation, infinite scrolling, autoplay, and immediate feedback mechanisms, thereby facilitating frequent and habitual use (Radesky et al., 2024; West et al., 2024). For adolescents, whose self-regulatory capacities are still developing, this media environment may increase the risk of uncontrolled or compensatory patterns of use (Montag et al., 2024; Shannon et al., 2022). Many users experience prolonged immersion and a weakened sense of time on platforms such as TikTok, YouTube Shorts, and Instagram Reels, and may gradually develop difficult-to-control use patterns in contexts of emotion regulation, social entertainment, and academic avoidance (Huang et al., 2022).

Problematic short-video use (PSVU) refers to an addiction-like pattern of short-video engagement characterized by impaired control, persistent immersion, withdrawal-like experiences, and impairment in daily functioning, and has increasingly emerged as an important adolescent public health concern (Li et al., 2025). According to the International Telecommunication Union (International Telecommunication Union, 2025), approximately 6 billion people worldwide were using the Internet in 2025, accounting for nearly three quarters of the global population. A cohort study published in JAMA involving 4,285 US adolescents further showed that, over a 4-year follow-up period, 31.3% of adolescents followed an increasing addictive social media use trajectory, and 24.6% followed an increasing addictive mobile phone use trajectory; high or increasing addictive screen-use trajectories were associated with poorer mental health, suicidal ideation, and suicidal behaviors (Xiao et al., 2025). PSVU may also pose risks to adolescents’ learning, sleep, emotional functioning, and daily adjustment (Jiang and Yoo, 2024; Zhan and Zhu, 2025). Given that the World Health Organization (2020) has incorporated the reduction of sedentary screen-based behavior into health-promotion recommendations for children and adolescents, clarifying the longitudinal associations of PSVU with academic adaptation and health-related behaviors has important public health and educational relevance.

Within the broader adolescent population, high school students represent a distinct educational and developmental group that warrants focused investigation. They are exposed not only to sustained academic demands and high-stakes educational evaluation but also to increasing autonomy in digital media use. During this stage, academic adaptation, physical exercise, and problematic digital media use may become especially intertwined. Therefore, the present study focused on Chinese high school students and examined the longitudinal associations between PSVU and their academic and health-related behaviors.

### 1.2 Learning burnout and problematic short-video use

Learning burnout (LB) refers to a state of emotional exhaustion, academic alienation, and reduced efficacy that emerges under sustained learning pressure and academic demands, and it represents an important manifestation of impaired academic adaptation among adolescents (Wu et al., 2010). In a study of 2,216 Chinese adolescents, Li et al. (2014) showed that academic alienation, physical and mental exhaustion, and reduced self-efficacy could reliably capture adolescents’ experiences of LB. Consistent with the primary conservation of resources (COR) framework, persistent academic demands can deplete cognitive, emotional, and social resources; when these demands exceed available resources, LB becomes more likely (Hobfoll, 1989; Salmela-Aro and Upadyaya, 2014). For high school students, continuous coursework, frequent academic evaluation, and limited recovery time may make the imbalance between learning demands and available psychological resources particularly salient.

When academic stress accumulates without sufficient recovery, high school students may experience emotional exhaustion, detachment from learning, and diminished academic efficacy. COR theory suggests that sustained resource loss may increase reliance on coping options that require little effort and provide immediate relief (Hobfoll, 1989). Short videos may therefore function as an accessible, escape-oriented form of regulation for students with elevated LB (Ye et al., 2025). Conversely, PSVU may precede higher LB by displacing sleep or academic recovery time and leaving fewer resources for sustained learning (Dewald et al., 2010; Zhao and Kou, 2024).

Cross-sectional studies have linked PSVU with LB, anxiety, impaired sleep, and daily functioning (Jiang and Yoo, 2024; Mao and Liao, 2025), but cannot establish temporal ordering. Their reciprocal association is therefore consistent with a possible COR loss spiral, although sleep disruption, time displacement, attentional depletion, and coping motives were not measured and remain plausible explanations only. Longitudinal analysis is needed to determine whether either direction, or both, operates over time. This question also has broader educational relevance because LB is associated with poorer academic performance, truancy or dropout, and adjustment difficulties (Bask and Salmela-Aro, 2013; Wang et al., 2015).

### 1.3 Physical exercise as a protective behavioral resource

Physical exercise (PE), as a low-cost and sustainable health behavior, may serve as a protective behavioral resource in the development of LB and PSVU (Biddle et al., 2019; Caspersen et al., 1985; Liu et al., 2026). Conservation of resources theory proposes that resource investment can generate additional resources and strengthen individuals’ capacity to cope with subsequent stressors (Hobfoll, 1989). In the high school context, PE may support physical recovery, emotional regulation, and peer interaction, thereby increasing the resources available for managing academic demands (Aguayo et al., 2019; Khosravi, 2021). Previous studies have similarly associated PE with lower LB through greater resilience, reduced stress, and improved emotional functioning (Deng et al., 2025; Fu et al., 2023). Thus, when a student engages in more PE than usual, subsequent LB may be lower.

PE may also be directly linked to subsequent PSVU. Temporal self-regulation theory suggests that behavioral choices reflect self-regulatory capacity, initiation costs, and the balance between immediate and delayed rewards (Hall, 2013; Hall and Fong, 2015). PE generally requires planning and effort and provides delayed benefits, whereas short videos offer immediate feedback at relatively little cost. PE may therefore be associated with lower subsequent PSVU by strengthening self-regulatory experiences, providing rewarding offline activities, and reducing reliance on immediate digital rewards (Li et al., 2021; Zhao et al., 2024).

These associations may also operate in the reverse direction. Higher LB may be associated with a reduced capacity to initiate and sustain PE, whereas PSVU may precede lower PE through possible displacement of after-school time and recovery (Zink et al., 2024). Within the COR framework, PE, LB, and PSVU may therefore be understood as an interconnected pattern of resource gain and loss (Zhao et al., 2024). Because changes in sleep, attention, time use, and self-regulatory resources were not directly measured, they are theoretical explanations rather than mechanisms established by the data. Longitudinal models are needed to test the direction and temporal consistency of these associations.

### 1.4 Learning burnout as a longitudinal indirect pathway

Within the COR framework developed above, LB may represent a contextually meaningful learning-adaptation pathway linking PE to subsequent PSVU (Chen et al., 2015; Hobfoll, 1989). Regular PE has been associated with cognitive and metacognitive functioning in children and adolescents (Álvarez-Bueno et al., 2017), which may be relevant to recovery and learning regulation. Lower LB may in turn be associated with less reliance on escape-oriented short-video use. Because cognition, recovery, learning regulation, and coping motives were not directly assessed, this account is presented as a theoretically plausible pathway rather than a demonstrated mechanism.

Previous cross-sectional research has found interrelations among PE, self-control, problematic digital media use, and LB (Du et al., 2025). However, concurrent associations cannot establish the temporal ordering of these variables. Therefore, the present study examined the longitudinal indirect pathway from T1 PE to T3 PSVU through T2 LB among Chinese high school students.

### 1.5 Current study

Existing studies on PE, LB, and PSVU have largely relied on cross-sectional designs, making it difficult to determine the temporal ordering and dynamic directionality among these three constructs. During high school, sustained academic demands, limited recovery time, and increasing autonomy in digital media use may create education-specific dynamic links among PE, LB, and PSVU. However, longitudinal evidence among high school students remains limited, and it is still unclear whether these constructs predict one another over time at the within-person level.

Moreover, the traditional cross-lagged panel model (CLPM) generally cannot distinguish stable between-person differences from within-person dynamic changes (Hamaker et al., 2015; Sorjonen and Melin, 2024). The RI-CLPM introduces random intercepts to decompose observed scores into stable between-person differences and within-person fluctuations and is therefore suitable for testing whether deviations from an individual’s usual level at one wave are associated with subsequent deviations at the next wave (Mulder and Hamaker, 2021). COR theory served as the primary framework for H1–H4, conceptualizing PE as a potential resource-gaining behavior and LB and PSVU as conditions associated with resource loss. Temporal self-regulation theory was used only as a complementary perspective for the direct PE–PSVU association.

Therefore, using three-wave longitudinal data from Chinese high school students, we used an RI-CLPM to examine stable between-person associations and within-person bidirectional cross-lagged relationships among PE, LB, and PSVU. We further tested the longitudinal indirect role of LB in the association between PE and subsequent PSVU. To complement the explanatory evidence from the RI-CLPM, we also conducted exploratory out-of-sample prediction of T3 PSVU using ridge regression and XGBoost. We examined the incremental predictive value of PE and LB beyond prior PSVU and used SHAP to interpret feature contributions (Chen and Guestrin, 2016; Hoerl and Kennard, 1970; Lundberg and Lee, 2017). Based on the theoretical framework and empirical evidence reviewed above, the following hypotheses were proposed:

*H1*: At the between-person level, PE would be negatively associated with LB and PSVU, whereas LB would be positively associated with PSVU.

*H2*: At the within-person level, PE would negatively predict subsequent LB and PSVU.

*H3*: At the within-person level, LB and PSVU would show positive reciprocal predictive associations, and both would negatively predict subsequent PE.

*H4*: At the within-person level, LB would play a longitudinal indirect role in the association between PE and subsequent PSVU.

## 2 Methods

### 2.1 Participants and procedure

This study adopted a three-wave longitudinal design. Data were collected at three time points: March 2025 (T1), September 2025 (T2), and March 2026 (T3), with a 6-month interval between adjacent waves. Participants were high school students from five schools in Yunnan Province, China. Questionnaire surveys were administered at the classroom level. At each wave, trained research assistants introduced the study purpose, questionnaire instructions, voluntary nature of participation, and procedures for confidentiality, pseudonymized longitudinal matching, and data protection before data collection. Students completed the questionnaires in class, and the completed questionnaires were collected on site. To enable longitudinal matching across waves, participants provided their student identification numbers. These identifiers were used solely for longitudinal matching and were handled confidentially during the matching process. After longitudinal matching was completed, the identifiers were removed from the analytic dataset, and subsequent analyses were conducted using de-identified data. The inclusion criteria were as follows: (1) being a high school student enrolled in one of the participating schools; (2) being able to independently understand and complete the questionnaire; (3) voluntarily agreeing to participate, with informed consent obtained from both the student and their legal guardian; and (4) providing identification information intended to permit longitudinal matching across waves. The exclusion criteria were as follows: (1) severe missing data on one or more core variables; (2) selecting the same response option for nearly all items; (3) clear evidence of random or patterned responding; (4) missing or unmatched longitudinal identification information; and (5) logically inconsistent demographic information across the three waves. At T1, 1,664 questionnaires constituted the baseline cohort after initial fieldwork screening. Of the 152 students who did not participate at T2, 97 were Grade 12 students from three classrooms who graduated between T1 and T2, whereas 55 were unavailable because of school transfer, absence, withdrawal, or other follow-up loss. A further 114 students completed T2 but did not participate at T3, resulting in 1,398 completed T3 questionnaires. During data cleaning and longitudinal matching, 28 questionnaires could not be reliably linked because of missing or inconsistent identifiers, 29 participants had an unavailable score for at least one core variable because of item-level missingness, and 14 were excluded because of patterned responding or inconsistent demographic information. The final complete three-wave analytic sample therefore comprised 1,327 students (79.75% of the valid T1 sample). Unavailable PE, LB, and PSVU scores numbered 2, 3, and 2 at T1; 3, 4, and 3 at T2; and 4, 5, and 6 at T3, respectively. These variable-level missing values were distinguished from wave-level nonparticipation and failure of longitudinal matching. The numbers of participants from Schools 1–5 were 327, 451, 382, 276, and 228 at T1; 300, 415, 363, 246, and 188 at T2; 267, 390, 339, 228, and 174 at T3; and 246, 380, 324, 219, and 158 in the final analytic sample, respectively. The corresponding T1-to-final retention rates were 75.23, 84.26, 84.82, 79.35, and 69.30%. The baseline sample included 800 Grade 10, 767 Grade 11, and 97 Grade 12 students; the corresponding final numbers were 680, 647, and 0. The complete loss of the baseline Grade 12 group reflected graduation between T1 and T2 rather than item-level missingness. The primary analyses were based on 1,327 participants with valid three-wave linkage and complete core-variable scores. Baseline T1 data remained available for the comparison between participants retained in and excluded from the final analytic sample. However, after de-identification, valid cross-wave linkage for excluded participants was not retained, and their incomplete longitudinal records could not be reconstructed at the individual level. Information on wave nonparticipation, linkage failures, and unavailable core-variable scores was therefore obtained from aggregate fieldwork and data-cleaning summaries. Consequently, no individually linked partial longitudinal dataset was available for FIML re-estimation, and FIML was not applied.

### 2.2 Measures

#### 2.2.1 Physical exercise

PE was assessed using the Physical Activity Rating Scale-3 (PARS-3; Liang, 1994). Although the established English name of the instrument contains the term “Physical Activity,” PARS-3 operationalizes exercise behavior through three components: exercise intensity, duration per session, and exercise frequency. Accordingly, throughout the present study, the construct assessed by PARS-3 is referred to as physical exercise (PE), whereas the broader term physical activity is retained only when referring to broader constructs in the cited literature or to official instrument and source titles.

The scale consists of three items assessing exercise intensity, duration per session, and exercise frequency. Example items include “What is the intensity of the physical exercise you usually engage in?”, “How long do you usually engage in the above exercise each time?”, and “How many times per month do you engage in the above exercise?” Each item is rated on a 5-point scale. The total PE score is calculated using the formula: exercise intensity × (exercise duration−1) × exercise frequency. Total scores range from 0 to 100, with higher scores indicating higher levels of PE. Scores of ≤19, 20–42, and ≥43 represent low, moderate, and high exercise levels, respectively.

PARS-3 is a formula-based behavioral composite rather than a conventional reflective scale: exercise intensity, duration, and frequency represent distinct components of exercise behavior and are not interchangeable indicators of a single latent factor. Accordingly, Cronbach’s alpha and reflective-factor measurement invariance were not evaluated. Longitudinal comparability was instead based on the use of identical item wording, response options, administration procedures, and scoring rules at T1, T2, and T3.

#### 2.2.2 Learning burnout

LB was assessed using the Adolescent Student Burnout Inventory (ASBI; Wu et al., 2010). The scale contains 16 items covering three dimensions: academic alienation, physical and mental exhaustion, and reduced self-efficacy. Representative items include “I think learning is meaningless” for academic alienation, “After a day of studying, I feel extremely exhausted” for physical and mental exhaustion, and “I do not experience a sense of achievement in learning” for reduced self-efficacy. Items are rated on a 5-point Likert scale ranging from 1 (“strongly disagree”) to 5 (“strongly agree”). After reverse scoring positively worded items, total scores are calculated by summing all items, with possible scores ranging from 16 to 80. Higher scores indicate higher levels of LB. In this study, Cronbach’s α values were 0.847, 0.845, and 0.841 at T1, T2, and T3, respectively, indicating good internal consistency across the three waves.

#### 2.2.3 Problematic short-video use

PSVU was assessed using the Short-Video Addiction Scale (Chen et al., 2025). The scale contains 17 items across four dimensions: time distortion, reality escape, withdrawal symptoms, and attention conflict. The first three dimensions each include four items, whereas the attention conflict dimension includes five items. Representative items include “I find that the time I spend watching short videos is always longer than I expected” for time distortion, “When I encounter a problem that I cannot solve, I watch short videos” for reality escape, “When I am unable to watch short videos, I become irritable or restless, but this disappears once I resume use” for withdrawal symptoms, and “Watching short videos makes me easily distracted when studying or working” for attention conflict. Items are rated on a 5-point Likert scale ranging from 1 (“strongly inconsistent with me”) to 5 (“strongly consistent with me”). Total scores range from 17 to 85, with higher scores indicating higher levels of PSVU. In the present study, Cronbach’s *α* values were 0.857, 0.862, and 0.858 at T1, T2, and T3, respectively, indicating good internal consistency across waves.

### 2.3 Covariates

Given that PE, LB, and PSVU may vary according to demographic and educational background characteristics among high school students, gender, academic performance, only-child status, parental educational attainment, and residence status were included as covariates. These covariates were used to predict the random intercepts of PE, LB, and PSVU, thereby controlling for stable between-person demographic differences. Gender was coded as 1 = boy and 2 = girl; only-child status was coded as 1 = yes and 2 = no; and residence status was coded as 1 = boarding and 2 = non-boarding. Academic performance was treated as an ordinal variable and coded as 1 = poor, 2 = below average, 3 = average, 4 = above average, and 5 = excellent. Parental educational attainment was also treated as an ordinal variable and coded as 1 = primary school or below, 2 = junior high school, 3 = senior high school, 4 = junior college/vocational school, and 5 = undergraduate degree or above.

### 2.4 Statistical analysis

Data were analyzed using SPSS 29.0, Mplus 8.3, and Python 3.12.13. Attrition bias was assessed by comparing T1 characteristics between the final three-wave sample (*n* = 1,327) and excluded participants (*n* = 337), using Welch’s t tests with standardized mean differences for continuous variables and Pearson’s *χ^2^* tests with Cramér’s *V* for categorical variables. The primary longitudinal analyses used the 1,327 complete three-wave matched cases. Although baseline T1 data permitted comparison between retained and excluded participants, valid cross-wave linkage for excluded cases was not retained after de-identification. These excluded records could therefore not be reconstructed as an individually linked partial longitudinal dataset for FIML re-estimation. Descriptive statistics and Pearson correlations were calculated. Harman’s single-factor test was conducted separately at each wave as a preliminary diagnostic but was not considered sufficient to exclude common method bias. Person-level unconditional random-intercept models were also estimated to quantify stable between-person and within-person variance.

Before structural modeling, longitudinal measurement invariance was examined separately for LB and PSVU using parcel-level CFA. At each wave, LB items were assigned to three fixed, nonoverlapping parcels comprising items 1–6, 7–11, and 12–16, and PSVU items to four parcels comprising items 1–5, 6–8, 9–11, and 12–17; no item was omitted or duplicated. Parcels were calculated as item means, with identical assignments across waves. Parcel-level indicators were used to evaluate aggregate score comparability while avoiding the extensive parameterization required by item-level three-wave models with 48 LB and 51 PSVU indicators. Configural, metric, and scalar invariance were tested sequentially, with ΔCFI ≤0.010 and ΔRMSEA ≤0.015 indicating invariance (Chen, 2007). These analyses support parcel-level longitudinal comparability but do not establish invariance of every item.

A traditional CLPM was first estimated as a reference, followed by an RI-CLPM examining longitudinal associations among PE, LB, and PSVU. The RI-CLPM separates stable between-person differences through random intercepts and estimates autoregressive, cross-lagged, and within-wave associations at the within-person level (Hamaker et al., 2015; Mulder and Hamaker, 2021). Core variables were entered as continuous observed scores, and the model was estimated using maximum likelihood. This specification preserved the established scale-scoring procedures and provided a parsimonious representation of the three variables across three waves. The preceding invariance analyses evaluated score comparability but did not introduce latent measurement models into the RI-CLPM; measurement error was therefore not separated from within-person fluctuations. Demographic and educational covariates predicted the random intercepts, and four school indicators were added in the school-adjusted sensitivity model. Standardized path estimates were reported. For the final RI-CLPM, 95% bias-corrected bootstrap confidence intervals for the standardized between-person, autoregressive, and cross-lagged estimates were obtained using 5,000 bootstrap samples.

Temporal invariance was evaluated using five nested RI-CLPMs: an unconstrained model (M1); models constraining corresponding cross-lagged paths (M2), autoregressive paths (M3), or T2–T3 within-wave residual covariances (M4) to equality; and a fully constrained model incorporating all three constraint sets (M5). Constraints were imposed on unstandardized parameters; standardized coefficients could therefore differ slightly across intervals because of wave-specific variances. Temporal invariance was evaluated primarily from acceptable absolute fit, ΔCFI ≤0.010, and ΔRMSEA ≤0.015, with *χ^2^* difference tests treated as complementary because of their sensitivity to minor discrepancies in large samples (Chen, 2007). In the retained model, STDYX-standardized estimates and 95% bias-corrected confidence intervals based on 5,000 bootstrap samples were reported for the principal autoregressive and cross-lagged paths and the longitudinal indirect association; the latter was additionally reported in unstandardized form. Acceptable fit was defined as CFI >0.90, RMSEA <0.08, and SRMR <0.08 (Hu and Bentler, 1999). All tests were two-tailed with *α* = 0.05.

Because only five schools were available, an exploratory school-clustered MLR model yielded unstable sandwich standard errors and was not used for inference. School-level robustness was instead assessed using the school-adjusted model and by re-estimating M5 after excluding each school in turn. Classroom-level clustering could not be examined because individual classroom identifiers were unavailable.

Supplementary predictive analyses used ridge regression and XGBoost to predict T3 PSVU (Chen and Guestrin, 2016; Hoerl and Kennard, 1970). These models were exploratory and were intended to complement the RI-CLPM analyses, not to validate causal interpretations of their longitudinal associations. For the T1-only and T1 + T2 predictor sets, baseline models included demographic variables and prior PSVU, whereas extended models additionally included PE and LB. The analytic sample contained no missing predictors or outcomes. Binary variables retained their two-level coding, academic performance and parental education retained their ordered 1–5 coding, and one-hot encoding was unnecessary. Predictors were standardized within the ridge pipeline; XGBoost used their original numeric scales.

Participants were stratified by empirical deciles of T3 PSVU and randomly allocated to development (80%, *n* = 1,061) and holdout sets (20%, *n* = 266). Nested cross-validation used five shuffled outer folds and four shuffled inner folds, with hyperparameters selected by minimizing cross-validated RMSE. Ridge α was selected from 25 logarithmically spaced values between 10^−3^ and 10^3^. For XGBoost, 24 random combinations were sampled for n_estimators (100, 200, 350, 500, 700), max_depth (2, 3, 4), learning_rate (0.02, 0.04, 0.06, 0.10), subsample and colsample_bytree (0.70, 0.85, 1.00), min_child_weight (1, 3, 5, 8), reg_alpha (0, 0.05, 0.20, 0.80), and reg_lambda (1, 3, 8, 15). All preprocessing and tuning were restricted to the relevant training folds. Final hyperparameters were selected by five-fold cross-validation within the full development set, after which the models were refitted on that set and evaluated on the untouched holdout set. A master seed of 20,260,704 governed allocation, fold shuffling, and tuning, with fold-specific seeds derived deterministically from it; bootstrap resampling used seed 20,260,803.

Holdout performance was evaluated using *R*^2^, RMSE, and MAE. Model differences were estimated from 2,000 paired bootstrap resamples, applying the same 266 resampled indices to both models and deriving percentile-based 95% confidence intervals. For leave-one-school-out validation, each school served once as the test set, while model fitting and four-fold grouped tuning used only the remaining schools. SHAP values were calculated in the holdout sample for the T1 + T2 extended XGBoost model and interpreted only as exploratory, model-specific feature attributions—not as evidence of incremental predictive value or causal effects (Lundberg and Lee, 2017).

## 3 Results

### 3.1 Common method Bias

Given the three-wave longitudinal design of this study, unrotated principal component analyses were conducted separately for all self-reported items at T1, T2, and T3 as a preliminary diagnostic of potential common method bias. Multiple factors with eigenvalues greater than 1 were extracted at each wave, and the first unrotated factor accounted for 17.55, 19.08, and 18.53% of the variance at T1, T2, and T3, respectively. These results indicate that no single factor dominated the covariance structure at any wave. However, Harman’s single-factor test is a limited diagnostic and cannot establish the absence or practical insignificance of common method bias (Podsakoff et al., 2003). Because PE, LB, and PSVU were all assessed by self-report from the same participants, shared-method variance and occasion-specific measurement error may still have contributed to the observed associations and should therefore be considered when interpreting the findings.

### 3.2 Sample attrition, sample characteristics, and preliminary analyses

The attrition analysis showed no significant differences between the final three-wave matched sample (*n* = 1,327) and participants not included in the final analytic sample (*n* = 337) in age, gender, other demographic characteristics, or baseline levels of T1 PE, LB, and PSVU (*p* = 0.116–0.741). All between-group effect sizes were small, with the largest absolute standardized mean difference being 0.073 and the largest Cramér’s V being 0.059. These findings suggest no clear evidence of systematic attrition bias based on the measured T1 baseline characteristics.

Table 1 presents the demographic characteristics and descriptive statistics of the unified analytic sample (*n* = 1,327). Panel A reports demographic characteristics, and Panel B reports the means and standard deviations of PE, LB, and PSVU across the three waves. The sample included 629 boys (47.40%) and 698 girls (52.60%); 777 participants (58.55%) were only children, and 1,043 participants (78.60%) were boarding students. Academic performance was mainly concentrated at the average level (43.48%), and parental educational attainment was most commonly senior high school (29.92%) or junior high school (24.87%). Across the three waves, PE showed relatively small mean-level variation. In contrast, LB exhibited a noticeable nonuniform temporal pattern, increasing from 47.67 at T1 to 50.24 at T2 and subsequently decreasing to 47.36 at T3. PSVU was 47.32 at T1, 48.11 at T2, and 45.56 at T3. These descriptive patterns indicate that the three constructs did not show uniformly stable mean levels across the study period.

**Table 1:** Demographic Characteristics and Descriptive Statistics of Study Variables for the Unified Analytical Sample.

Panel A. Demographic characteristics		
Variables	*N*	%
Gender		
Boys	629	47.40
Girls	698	52.60
Academic performance		
Poor	122	9.19
Below average	326	24.57
Average	577	43.48
Above average	249	18.76
Excellent	53	3.99
Only-child status		
Yes	777	58.55
No	550	41.45
Parental education		
Primary school	129	9.72
Junior high school	330	24.87
Senior high school	397	29.92
Junior college/vocational school	294	22.16
Undergraduate degree or above	177	13.34
Residence		
Boarding	1,043	78.60
Non-boarding	284	21.40

Panel B. Descriptive statistics of study variables		
Variables	*M*	*SD*
PE T1	26.22	13.66
LB T1	47.67	8.74
PSVU T1	47.32	9.12
PE T2	27.10	13.49
LB T2	50.24	8.64
PSVU T2	48.11	9.35
PE T3	27.02	13.54
LB T3	47.36	8.64
PSVU T3	45.56	9.18

*N* = 1,327. PE, physical exercise; LB, learning burnout; PSVU, problematic short-video use; M, mean; SD, standard deviation.

As shown in Figure 1, PE was significantly and negatively correlated with both LB and PSVU within and across waves. The within-wave correlations ranged from *r* = −0.217 to −0.279 for PE–LB and from *r* = −0.143 to −0.264 for PE–PSVU. In contrast, LB was significantly and positively correlated with PSVU across all three waves, with within-wave correlations ranging from *r* = 0.247 to 0.343. The constructs also showed moderate to strong positive stability across adjacent waves. The cross-wave stability correlations ranged from *r* = 0.532 to 0.627 for PE, from *r* = 0.380 to 0.535 for LB, and from *r* = 0.434 to 0.573 for PSVU. Overall, the correlation heatmap indicates that PE was primarily negatively associated with LB and PSVU, whereas LB was primarily positively associated with PSVU. These zero-order correlations provide preliminary support for further distinguishing stable between-person differences from within-person dynamic processes.

**Figure 1:**
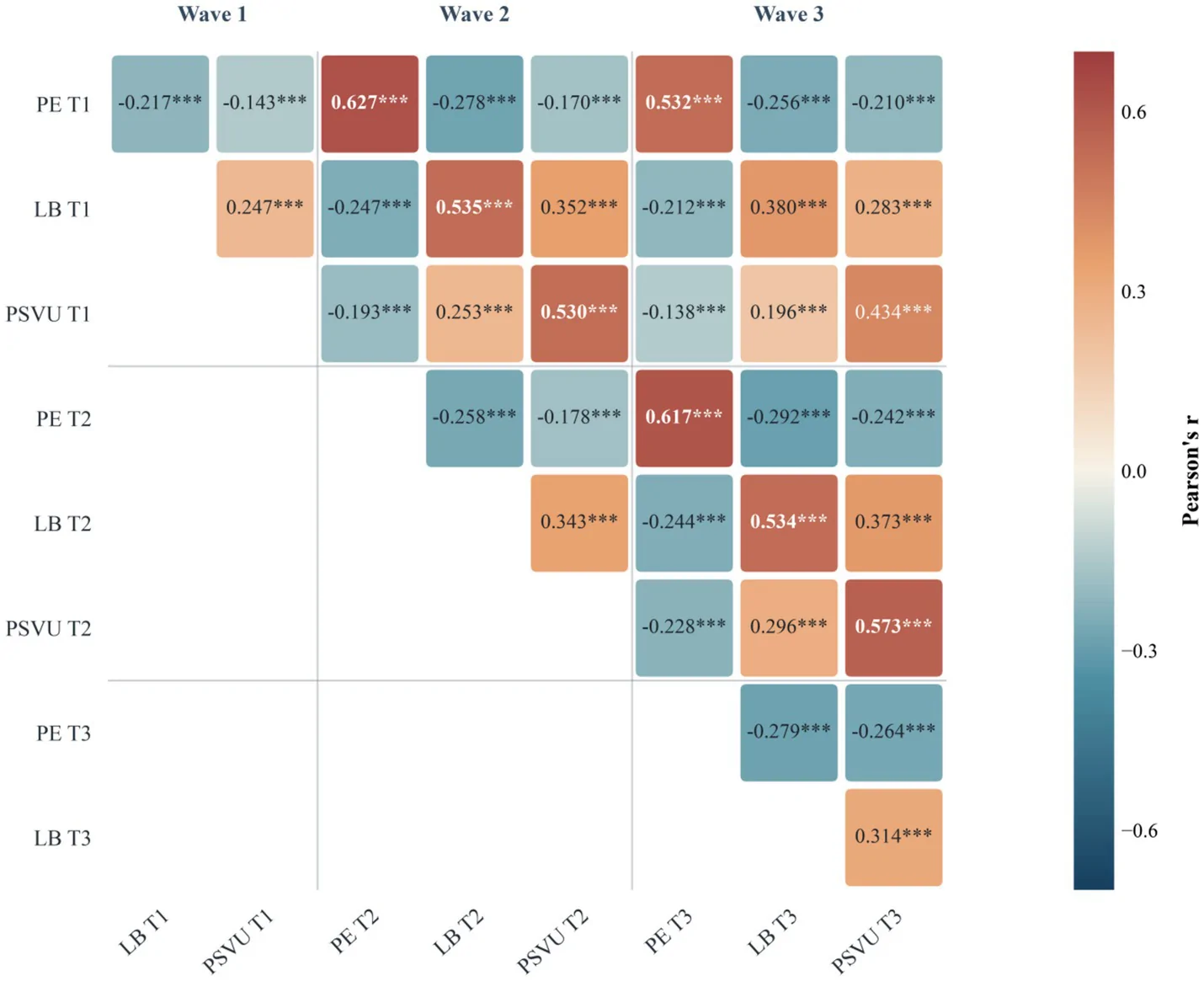
Correlation heatmap of PE, LB, and PSVU across three waves. ^***^p < 0.001.

In addition, based on the three repeated measurements, the individual-level ICCs for PE, LB, and PSVU were 0.592, 0.483, and 0.511, respectively. Specifically, 59.2% of the total variance in PE was attributable to stable between-person differences, whereas 40.8% was attributable to within-person fluctuations over time. For LB, 48.3% of the total variance was attributable to between-person differences and 51.7% to within-person fluctuations. For PSVU, 51.1% of the total variance was attributable to between-person differences and 48.9% to within-person fluctuations. These results indicate that all three variables contained both substantial stable between-person differences and meaningful within-person dynamic variation, providing empirical support for the use of RI-CLPM to separate between-person and within-person effects.

### 3.3 Measurement model and longitudinal invariance

Before testing the structural relations, parcel-level longitudinal measurement invariance was examined for LB and PSVU (Table 2). The configural models showed acceptable fit for both constructs, indicating that the same parcel-level measurement structure was retained across waves. Equality constraints on corresponding parcel loadings produced only small changes in fit, supporting metric invariance. Further constraining corresponding parcel intercepts likewise did not substantially deteriorate model fit, supporting scalar invariance at the parcel level. These results indicate that the aggregate parcel indicators showed adequate longitudinal comparability across the three waves. However, because the analyses were conducted using parcels, they should not be interpreted as demonstrating longitudinal invariance of every individual item or as accounting for measurement error in the subsequent observed-score RI-CLPM.

**Table 2:** Longitudinal measurement invariance of LB and PSVU.

Construct	Model	*CFI*	*RMSEA*	*ΔCFI*	*ΔRMSEA*
LB	Configural invariance	0.956	0.045		
	Metric invariance	0.954	0.042	−0.002	−0.003
	Scalar invariance	0.951	0.040	−0.003	−0.002
PSVU	Configural invariance	0.984	0.044		
	Metric invariance	0.981	0.044	−0.003	0
	Scalar invariance	0.979	0.046	−0.002	0.002

CFI, comparative fit index; RMSEA, root mean square error of approximation. ΔCFI and ΔRMSEA were calculated relative to the preceding, less constrained model. Configural invariance tested whether the same parcel-level structure was retained across waves; metric invariance constrained corresponding parcel loadings to equality, whereas scalar invariance additionally constrained corresponding parcel intercepts to equality. Changes in model fit were within the prespecified ranges for both LB and PSVU, supporting configural, metric, and scalar invariance of the parcel indicators across the three waves. These results concern aggregate parcel-level comparability and do not establish invariance of every individual item.

### 3.4 Model comparisons

To examine the suitability of RI-CLPM relative to the traditional CLPM, a traditional CLPM including covariates was first estimated. The results showed that the traditional CLPM had an acceptable fit, *χ^2^* (24) = 174.09, CFI = 0.960, RMSEA = 0.069, and SRMR = 0.036. The path results preliminarily indicated that PE, LB, and PSVU all showed significant cross-time stability and bidirectional lagged associations. However, because CLPM cannot distinguish stable between-person differences from within-person dynamic fluctuations, it may misinterpret trait-like differences as within-person lagged effects. Therefore, the CLPM results were interpreted only as a reference model. The corresponding traditional CLPM is presented in Figure 2.

**Figure 2:**
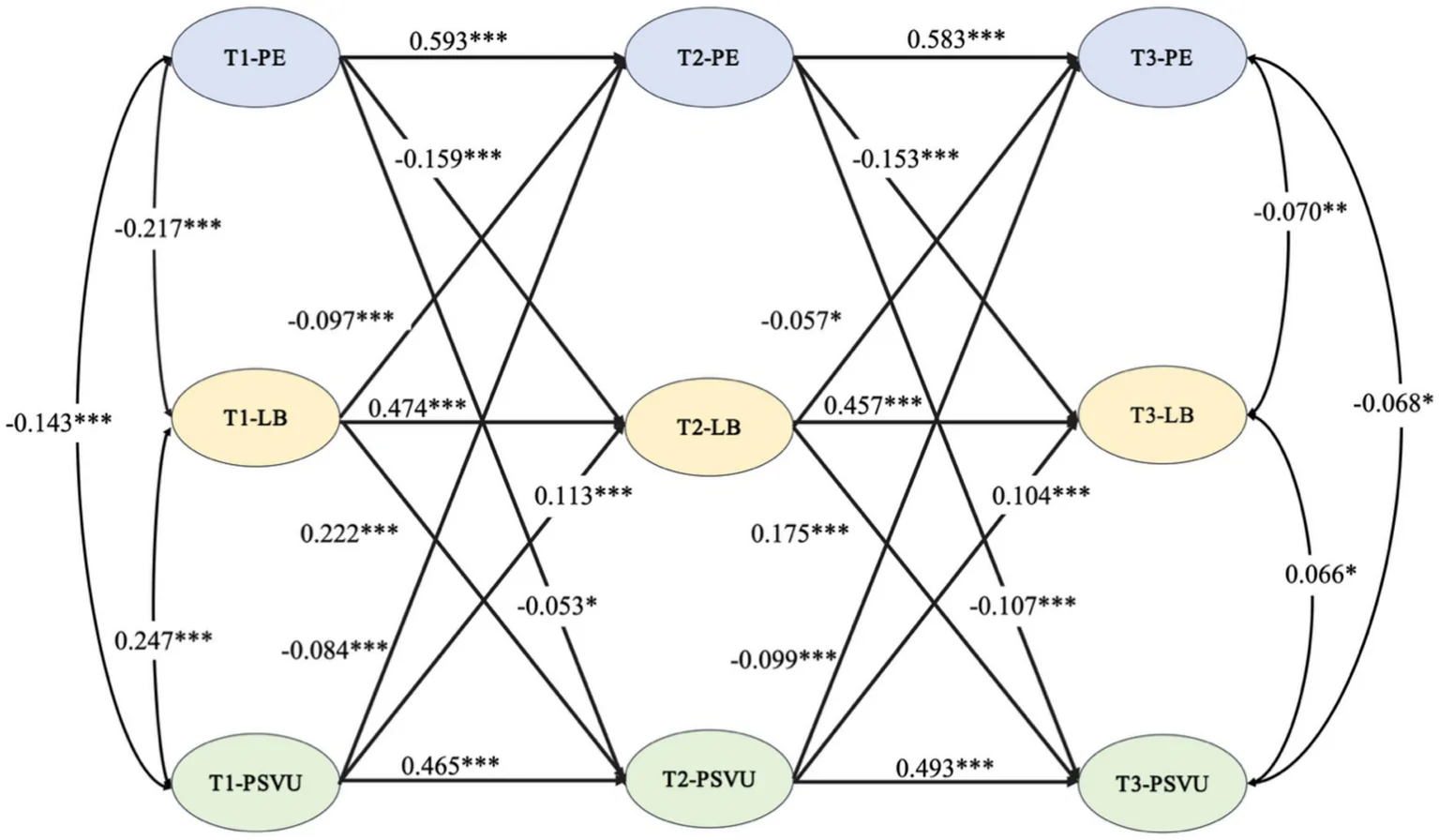
Traditional cross-lagged panel model of PE, LB, and PSVU across three waves. PE = physical exercise; LB = learning burnout; PSVU = problematic short-video use; T = time point. Standardized coefficients are shown. Only significant autoregressive, cross-lagged paths, and within-wave correlations are displayed. Covariate paths are omitted for clarity. ^*^p < 0.05, ^**^p < 0.01, ^***^p < 0.001.

Subsequently, temporal invariance of the RI-CLPM parameters was examined (Table 3). The unconstrained model showed good fit, *χ^2^* (33) = 38.49, *p* = 0.235, CFI = 0.989, RMSEA = 0.011, and SRMR = 0.013. Constraining the cross-lagged paths (M2) or autoregressive paths (M3) produced negligible changes in approximate fit. Although the *χ^2^* difference between M4 and M1 was significant, Δ*χ^2^* (3) = 9.64, *p* = 0.022, the changes in CFI and RMSEA were small (ΔCFI = −0.002; ΔRMSEA = 0.005), and M4 retained good absolute fit. This result was therefore interpreted as a limited practical deterioration rather than sufficient evidence against temporal invariance. The fully constrained M5 also showed good fit, *χ^2^* (45) = 53.60, *p* = 0.178, CFI = 0.988, RMSEA = 0.012, and SRMR = 0.016, and did not differ significantly from M1, Δ*χ^2^* (12) = 15.11, *p* = 0.235, ΔCFI = −0.001, and ΔRMSEA = 0.001. Accordingly, M5 was retained as the more parsimonious primary model. All equality constraints were imposed on unstandardized parameters; the standardized estimates reported below may therefore differ slightly across intervals.

**Table 3:** Model fits and comparisons for random intercept Cross-Lagged panel models.

Model fits						Model comparisons				
Models	*χ^2^*	df	CFI	RMSEA	SRMR	Comparisons	Δχ^2^	Δdf	ΔCFI	ΔRMSEA
M1	38.49	33	0.989	0.011	0.013					
M2	44.44	39	0.989	0.010	0.014	M2–M1	5.94	6	0	−0.001
M3	39.59	36	0.989	0.009	0.013	M3–M1	1.10	3	0	−0.002
M4	48.13	36	0.987	0.016	0.015	M4–M1	9.64	3	−0.002	0.005
M5	53.60	45	0.988	0.012	0.016	M5–M1	15.11	12	−0.001	0.001

M1, unconstrained baseline model; M2, model constraining corresponding cross-lagged paths to equality across intervals; M3, model constraining corresponding autoregressive paths to equality; M4, model constraining corresponding T2 and T3 within-wave residual covariances to equality; M5, model incorporating all three sets of equality constraints. Equality constraints were imposed on unstandardized parameters. ΔCFI and ΔRMSEA were calculated relative to M1. Temporal invariance was evaluated primarily from acceptable absolute fit, ΔCFI ≤0.010, and ΔRMSEA ≤0.015; χ^2^ difference tests were treated as complementary evidence.

### 3.5 Final RI-CLPM results

STDYX-standardized estimates and their 95% bias-corrected bootstrap confidence intervals are reported in Table 4, while Figure 3 provides a graphical summary of the principal paths. The equality constraints in M5 were imposed on corresponding unstandardized parameters; therefore, the reported standardized coefficients may differ slightly across intervals because they incorporate wave-specific variances. At the between-person level, RI-PE was significantly and negatively associated with both RI-LB (*β* = −0.440, *p* = 0.001) and RI-PSVU (*β* = −0.240, *p* = 0.002), whereas RI-LB was significantly and positively associated with RI-PSVU (*β* = 0.461, *p* = 0.001). These findings indicate that adolescents with higher long-term levels of PE generally had lower levels of LB and PSVU, whereas those with higher long-term levels of LB tended to show higher levels of PSVU.

**Table 4:** Standardized estimates of the final time-invariant RI-CLPM.

Effect level	Path	T1 → T2 *β* [95% BC bootstrap CI]	T2 → T3 *β* [95% BC bootstrap CI]
Between-person associations	RI-PE ↔ RI-LB	−0.440 [−0.639, −0.214]	
	RI-PE ↔ RI-PSVU	−0.240 [−0.370, −0.065]	
	RI-LB ↔ RI-PSVU	0.461 [0.129, 0.647]	
Autoregressive effects	PE → PE	0.245 [0.155, 0.337]	0.236 [0.139, 0.335]
	LB → LB	0.354 [0.273, 0.445]	0.344 [0.259, 0.431]
	PSVU → PSVU	0.245 [0.169, 0.319]	0.255 [0.167, 0.336]
Cross-lagged effects	PE → LB	−0.147 [−0.216, −0.080]	−0.142 [−0.206, −0.079]
	PE → PSVU	−0.105 [−0.177, −0.035]	−0.112 [−0.176, −0.034]
	LB → PE	−0.087 [−0.164, −0.012]	−0.084 [−0.157, −0.012]
	LB → PSVU	0.239 [0.168, 0.310]	0.245 [0.171, 0.307]
	PSVU → PE	−0.158 [−0.229, −0.089]	−0.169 [−0.231, −0.089]
	PSVU → LB	0.104 [0.043, 0.167]	0.104 [0.043, 0.169]

*β*, STDYX-standardized estimate; BC, bias-corrected. Confidence intervals were based on 5,000 bootstrap samples. Between-person associations are not interval-specific and are therefore reported only once. Equality constraints were imposed on the corresponding unstandardized parameters; standardized estimates may differ slightly across intervals because of wave-specific variances.

**Figure 3:**
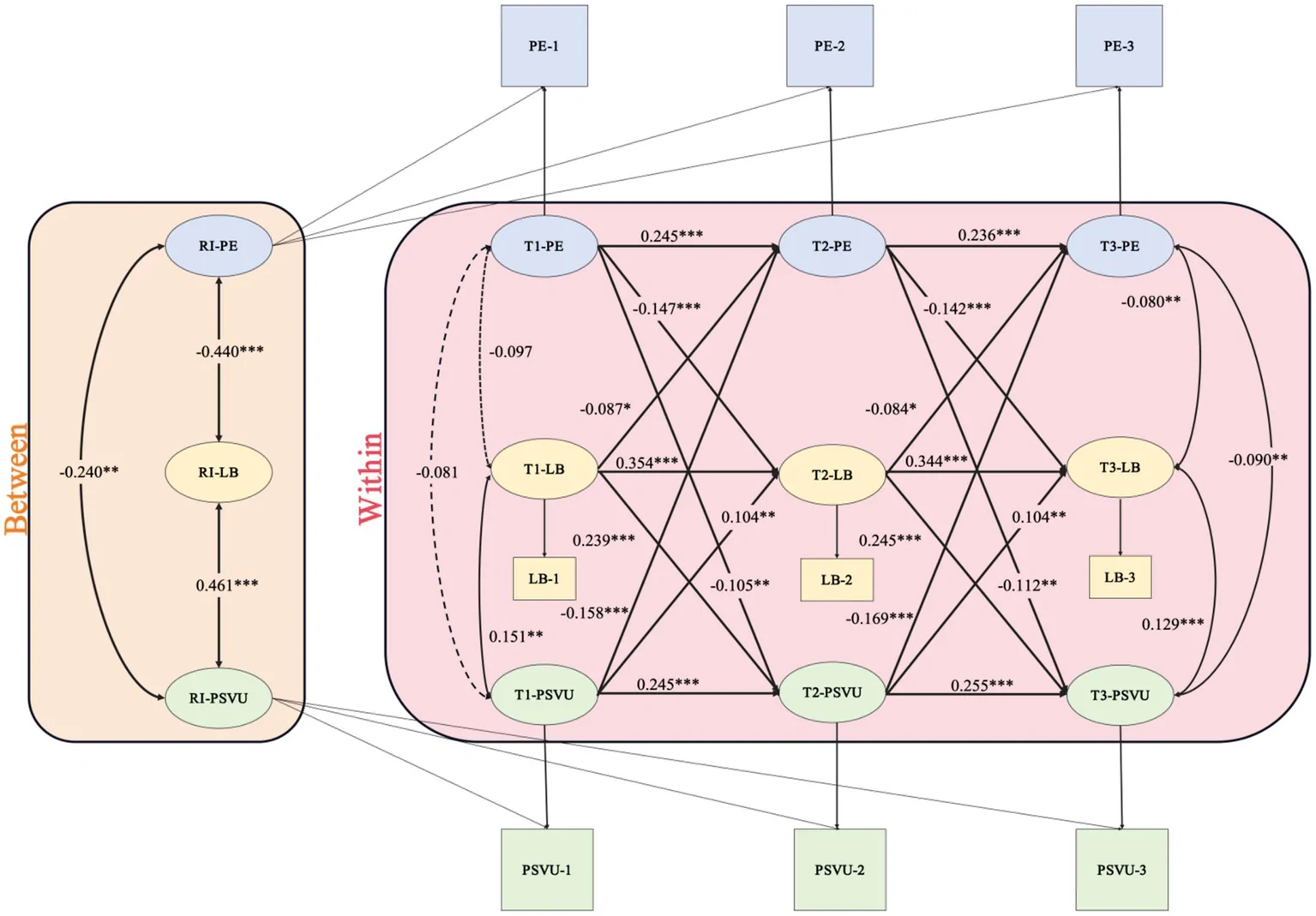
Final time-invariant RI-CLPM of PE, LB, and PSVU across three waves. PE = physical exercise; LB = learning burnout; PSVU = problematic short-video use. Equality constraints in M5 were imposed on corresponding unstandardized autoregressive and cross-lagged coefficients and T2–T3 within-wave residual covariances. STDYX-standardized coefficients are displayed and may therefore differ slightly across intervals because they depend on wave-specific variances. Solid and dashed paths indicate significant and nonsignificant effects, respectively. Factor loadings from the observed variables to the random intercepts and within-person latent factors were fixed at 1. Covariate paths are omitted for clarity. *p < 0.05, **p < 0.01, ***p < 0.001.

At the within-person level, PE, LB, and PSVU showed significant positive autoregressive effects in M5. The constrained cross-lagged estimates indicated that higher-than-usual PE was associated with lower subsequent LB and PSVU; higher-than-usual LB was associated with lower subsequent PE and higher subsequent PSVU; and higher-than-usual PSVU was associated with lower subsequent PE and higher subsequent LB. As shown in Supplementary Table S1, all nine autoregressive and cross-lagged paths retained the same direction in the unconstrained M1, and seven were statistically significant in both intervals. Two comparatively weak paths showed interval-specific uncertainty: PE → PSVU was nonsignificant from T1 to T2 (*β* = −0.041, *p* = 0.382) but significant from T2 to T3 (*β* = −0.132, *p* = 0.004), whereas LB → PE was significant from T1 to T2 (*β* = −0.103, *p* = 0.016) but nonsignificant from T2 to T3 (*β* = −0.054, *p* = 0.302). Importantly, differences in statistical significance across intervals do not themselves establish significant differences between the corresponding coefficients, and constraining the cross-lagged paths jointly did not significantly worsen model fit. M5 was therefore retained as a parsimonious representation of the overall temporal pattern, while the interval-specific evidence for PE → PSVU and LB → PE should be interpreted cautiously. These findings represent within-person longitudinal associations rather than causal effects.

School-level sensitivity analyses broadly supported the primary RI-CLPM. The school-adjusted M5 showed excellent fit, *χ^2^* (69) = 87.04, CFI = 0.995, TLI = 0.992, RMSEA = 0.014, and SRMR = 0.017. All nine autoregressive and cross-lagged paths retained their directions and statistical significance, and their model-based standardized SEs differed from those of the primary model by no more than 0.001. In the leave-one-school-out analyses, all path directions remained stable, and seven of the nine paths remained significant in every re-estimated model. The two exceptions were the comparatively small LB → PE path when School 3 was excluded (*p* = 0.064) and the PE → PSVU path when School 1 was excluded (*p* = 0.051).

### 3.6 Longitudinal indirect association through learning burnout

The longitudinal indirect association was examined in the final time-invariant RI-CLPM. T1 PE was negatively associated with T2 LB (*B* = −0.007, *β* = −0.147, 95% bias-corrected bootstrap CI for *β* [−0.216, −0.080]), which was positively associated with T3 PSVU (*B* = 0.222, *β* = 0.240, 95% CI [0.171, 0.307]). The indirect association was statistically significant but small (*B* = −0.002, 95% CI [−0.003, −0.001], *p* < 0.001; *β* = −0.035, 95% CI [−0.057, −0.018]; Table 5). It remained significant after adjustment for school fixed effects (*B* = −0.002, 95% CI [−0.002, −0.001], *p* < 0.001). These findings support a small, temporally ordered longitudinal indirect association rather than causal mediation.

**Table 5:** Component paths and longitudinal indirect association in the final time-invariant RI-CLPM.

Estimate	*B*	*SE*	95% BC bootstrap CI	*β*	95% BC bootstrap CI
T1 PE → T2 LB	−0.007	0.002	[−0.010, −0.004]	−0.147	[−0.216, −0.080]
T2 LB → T3 PSVU	0.222	0.032	[0.158, 0.284]	0.245	[0.171, 0.307]
T1 PE → T2 LB → T3 PSVU	−0.002	0.0004	[−0.003, −0.001]	−0.035	[−0.057, −0.018]

*B* = unstandardized estimate; *β*, STDYX-standardized estimate; BC bootstrap CI, bias-corrected bootstrap confidence interval. Confidence intervals were based on 5,000 bootstrap samples.

### 3.7 Exploratory machine-learning results

The exploratory machine-learning analysis examined the incremental predictive value of PE and LB for T3 PSVU from a student-level out-of-sample prediction perspective. As shown in Table 6, when only T1 information was included, the models achieved holdout test-set *R^2^* values ranging from 0.142 to 0.155. After T2 information was added, the holdout test-set *R^2^* increased to 0.288–0.311. Among the original student-level holdout models, the T1 + T2 baseline XGBoost model showed the best predictive performance, with an *R^2^* of 0.311, an RMSE of 7.502, and an MAE of 6.021.

**Table 6:** Cross-validated and student-level holdout predictive performance of supplementary machine-learning models predicting T3 PSVU.

Predictor set	Model	CV *R*^2^	CV RMSE	CV MAE	Holdout *R*^2^	Holdout RMSE	Holdout MAE
T1 baseline	Ridge	0.184 ± 0.029	8.320 ± 0.399	6.649 ± 0.299	0.142	8.368	6.726
T1 baseline	XGBoost	0.180 ± 0.022	8.344 ± 0.449	6.683 ± 0.359	0.155	8.307	6.677
T1 extended	Ridge	0.236 ± 0.027	8.055 ± 0.467	6.429 ± 0.397	0.151	8.328	6.605
T1 extended	XGBoost	0.234 ± 0.032	8.063 ± 0.480	6.464 ± 0.423	0.155	8.307	6.639
T1 + T2 baseline	Ridge	0.348 ± 0.053	7.424 ± 0.347	5.911 ± 0.304	0.289	7.618	6.108
T1 + T2 baseline	XGBoost	0.334 ± 0.043	7.510 ± 0.320	5.992 ± 0.285	0.311	7.502	6.021
T1 + T2 extended	Ridge	0.395 ± 0.047	7.155 ± 0.352	5.673 ± 0.313	0.288	7.626	6.093
T1 + T2 extended	XGBoost	0.377 ± 0.047	7.262 ± 0.302	5.789 ± 0.281	0.294	7.591	6.101

CV results are presented as mean ± standard deviation across the five outer folds of nested cross-validation. All preprocessing and hyperparameter selection were confined to the corresponding training data, and the holdout set was used only after final model refitting. The development and final holdout samples included 1,061 and 266 participants, respectively. The T1 baseline models included demographic covariates and T1 PSVU; the T1 extended models additionally included T1 PE and T1 LB. The T1 + T2 baseline models included demographic covariates and PSVU measured at T1 and T2; the corresponding extended models additionally included PE and LB measured at T1 and T2. Higher *R^2^* and lower RMSE and MAE indicate better predictive performance. PE, physical exercise; LB, learning burnout; PSVU, problematic short-video use; CV, cross-validation; RMSE, root mean square error; MAE, mean absolute error.

However, adding PE and LB to models that already included prior PSVU and demographic variables did not consistently improve predictive performance in the holdout test set. For the T1 models, the increase in *R*^2^ from the baseline to the extended model was 0.008 for Ridge regression, 95% CI [−0.043, 0.059], and approximately 0 for XGBoost, 95% CI [−0.057, 0.051]. For the T1 + T2 models, the corresponding increases were −0.001, 95% CI [−0.044, 0.039], and −0.016, 95% CI [−0.062, 0.026], respectively. In the T1 + T2 extended models, the increase in *R*^2^ for XGBoost relative to Ridge regression was 0.006, 95% CI [−0.014, 0.028]. Because all confidence intervals included zero, these results indicate that adding PE and LB did not provide stable incremental predictive value beyond prior PSVU and demographic variables. They also suggest that XGBoost did not show a clear predictive advantage over Ridge regression in this dataset.

To evaluate predictive performance without same-school overlap between model development and testing, a leave-one-school-out sensitivity analysis was conducted. All school-specific test folds yielded positive *R*^2^ values, ranging from 0.116 to 0.409 across model specifications. For the T1-only predictor sets, pooled *R*^2^ values ranged from 0.177 to 0.231; after T2 predictors were included, they ranged from 0.337 to 0.382. The T1 + T2 extended Ridge model showed the best pooled school-grouped performance (*R*^2^ = 0.382, RMSE = 7.236, MAE = 5.746), followed by the T1 + T2 extended XGBoost model (*R*^2^ = 0.365, RMSE = 7.335, MAE = 5.850). Although the extended models yielded higher point estimates than their corresponding baseline models, no inferential comparisons were made because only five school folds were available. These differences should therefore not be interpreted as evidence of stable incremental predictive value.

Exploratory SHAP analysis of the T1 + T2 extended XGBoost model showed that T2 PSVU accounted for 45.5% of the total absolute SHAP contribution, followed by T2 LB, T1 PSVU, and T2 PE, which accounted for 17.5, 17.2, and 11.2%, respectively (Figure 4). T1 LB, T1 PE, and the demographic covariates made smaller contributions. These values describe the allocation of predictive attribution within the fitted extended model. Because adding PE and LB did not reliably improve holdout performance over the corresponding baseline model, their SHAP contributions should not be interpreted as evidence of incremental predictive value beyond prior PSVU and demographic variables.

**Figure 4:**
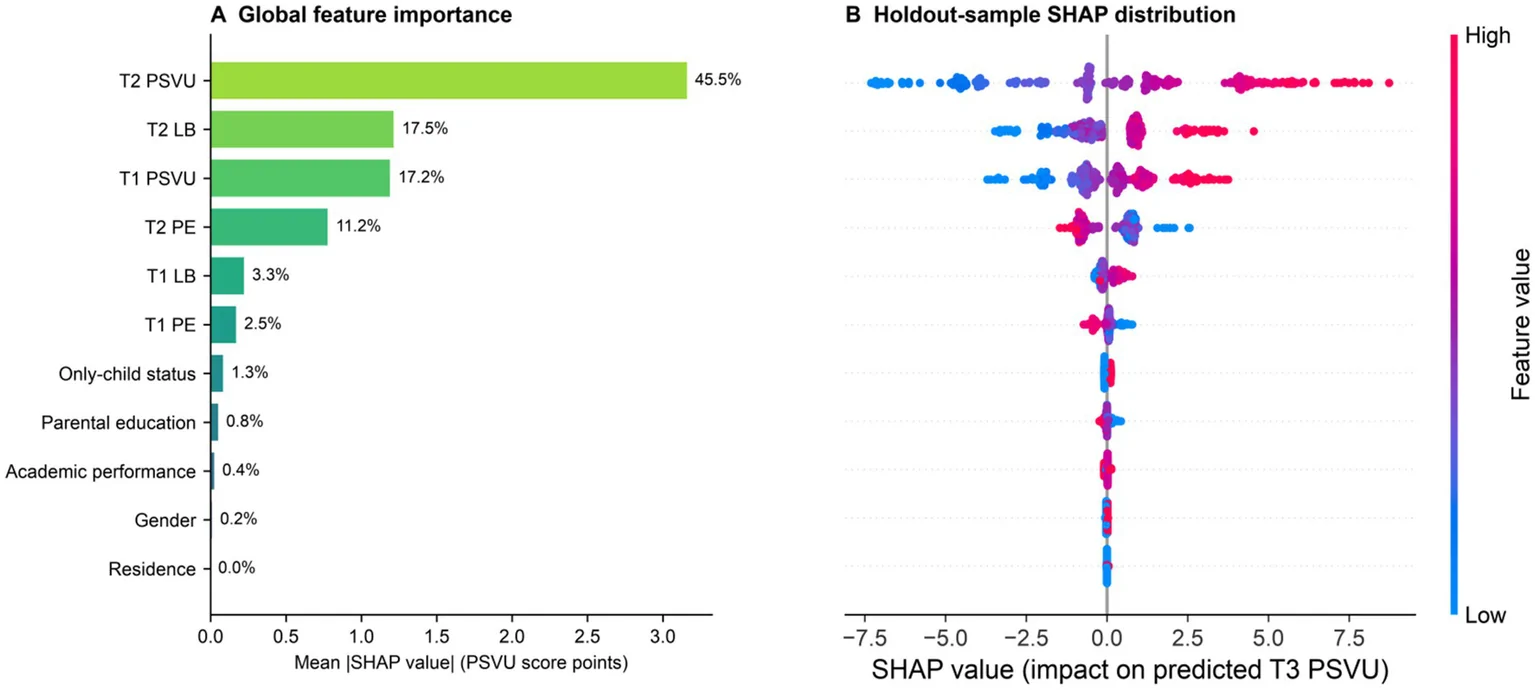
SHAP interpretation of the T1 + T2 extended XGBoost model. Panel **A** presents global feature attribution based on mean absolute SHAP values, and Panel **B** presents the distribution and direction of SHAP values in the holdout sample. Red and blue points represent higher and lower feature values, respectively; positive SHAP values indicate higher predicted T3 PSVU. SHAP values are exploratory, model-specific attributions within the T1 + T2 extended XGBoost model and do not establish incremental predictive value beyond the baseline model or causal effects.

## 4 Discussion

Based on three-wave longitudinal data from Chinese high school students, this study used an RI-CLPM to examine the longitudinal associations among PE, LB, and PSVU at both the between- and within-person levels. The constrained M5 supported an overall pattern in which higher-than-usual PE was associated with lower subsequent LB and PSVU, LB and PSVU showed positive reciprocal associations, and higher-than-usual LB and PSVU were associated with lower subsequent PE. The unconstrained M1 retained the same directional pattern but revealed interval-specific uncertainty in the comparatively weak PE → PSVU and LB → PE paths. A significant but small longitudinal indirect association from T1 PE to T3 PSVU through T2 LB was also observed. These findings extend previous cross-sectional evidence by showing that fluctuations in PE, LB, and PSVU within the same individual are prospectively related across waves. Consistent with prior evidence that LB predicts later compulsive Internet use and academic impairment (Liou et al., 2022), the present results further suggest that LB may represent one longitudinal pathway linking PE to subsequent PSVU. Importantly, however, although the RI-CLPM separates stable between-person differences from within-person fluctuations, it does not eliminate confounding from unmeasured factors that vary over time (Mund et al., 2021). Accordingly, the within-person estimates should be interpreted as temporally ordered longitudinal associations rather than causal effects. Compared with the traditional CLPM, the principal value of the RI-CLPM lies in examining whether deviations from an individual’s own usual level are associated with subsequent deviations in related outcomes (Hamaker et al., 2015).

### 4.1 Longitudinal relationship between physical exercise and problematic short-video use

This study found that PE negatively predicted subsequent PSVU after stable between-person differences were controlled, supporting H2. This finding is important not because students with higher overall PE tend to report lower PSVU, but because it indicates a within-person association. When the same student engaged in more PE than their usual level at a given wave, the severity of subsequent PSVU tended to be lower. This within-person association is less susceptible to confounding by stable personality traits, family background, or long-term lifestyle differences, but may still be influenced by unmeasured time-varying factors. Therefore, the finding should be interpreted as evidence that higher-than-usual PE is associated with lower subsequent problematic use rather than as proof of a causal effect (Li et al., 2025).

Consistent with the COR account and the complementary temporal self-regulation perspective outlined above, the PE–PSVU association may reflect differences in resource demands, initiation costs, and the relative value of immediate and delayed outcomes (Hobfoll, 1989; Hall, 2013; Hall and Fong, 2015). Short-video use provides immediate feedback at relatively little initiation cost, whereas PE requires planning, physical effort, and acceptance of more delayed benefits. Adolescents may be particularly responsive to novelty, immediate feedback, and social rewards (Chein et al., 2011; Galván, 2010; Somerville et al., 2010). Peer and platform feedback may further increase the reinforcing value of short-video content (Albert et al., 2013; Sherman et al., 2018). This combination may make short videos especially attractive during periods of academic fatigue or reduced self-regulatory resources, although reward sensitivity and self-regulatory capacity were not directly assessed in the present study.

From the same perspective, PE may provide a resource-enhancing alternative through physical recovery, offline reward, competence experiences, and social interaction. Previous longitudinal evidence has similarly linked PE and self-control with lower subsequent problematic mobile-phone use (Zhao et al., 2024), while other research has associated PE with lower psychological distress and problematic mobile-phone use (Li et al., 2021). These findings provide indirect support for a resource-based interpretation of the PE–PSVU association. However, because the present study did not directly measure executive control, reward processing, offline reward, or actual short-video viewing duration, these processes should be regarded as plausible explanations rather than mechanisms demonstrated by the data.

This study also found that PSVU negatively predicted subsequent PE, supporting H3. The measured characteristics of PSVU offer several possible explanations for this direction of association. Time distortion may interfere with students’ planning of after-school activities, attention conflict may weaken the execution of exercise plans, and reality escape or withdrawal discomfort may increase reliance on readily accessible digital activities. Previous studies have associated problematic digital-media use with sleep disturbance and less favorable health-behavior patterns (Chen and Wu, 2021; Zhang et al., 2023). Research using broader physical-activity measures has also linked higher screen exposure with lower activity participation and poorer sleep recovery (Stiglic and Viner, 2019; Zablotsky et al., 2025). These findings provide relevant contextual evidence, but they should not be treated as construct-equivalent evidence for PARS-3-assessed PE. Moreover, sleep disruption, time displacement, attentional depletion, and effort valuation were not directly measured; they remain possible explanations rather than established pathways.

Taken together, M5 supported an overall reciprocal association between PE and PSVU, but M1 provided stronger interval-specific evidence for PSVU → PE, which was significant in both intervals, than for PE → PSVU, which was significant only from T2 to T3. The findings therefore support a focused resource-based account in which PE and PSVU may be longitudinally interrelated, while not establishing a temporally uniform or causal feedback cycle. They may inform the identification of candidate behavioral targets for future intervention research, but they do not demonstrate that increasing PE will reduce PSVU or that reducing PSVU will increase PE. Whether changing either behavior produces subsequent changes in the other requires direct experimental or quasi-experimental evidence.

### 4.2 Dynamic longitudinal associations and the longitudinal indirect role of learning burnout

The RI-CLPM results indicated that PE, LB, and PSVU were not independent of one another. At the between-person level, high school students with higher long-term levels of PE generally showed lower levels of LB and PSVU, whereas those with higher long-term levels of LB tended to report higher levels of PSVU, supporting H1. The within-person results further showed that when the same student’s PE was higher than their usual level at one wave, their LB was relatively lower at the next wave. Conversely, when LB was higher than usual, subsequent PSVU was relatively higher. In addition, PSVU positively predicted subsequent LB, and both LB and PSVU negatively predicted later PE. Taken together, the constrained M5 supported H2 and H3 at the overall time-invariant level and suggested a reciprocal pattern of temporally ordered within-person associations rather than a simple unidirectional sequence. However, the unconstrained M1 retained the same path directions but showed interval-specific uncertainty for the comparatively weak PE → PSVU and LB → PE paths. Because unmeasured time-varying confounding cannot be excluded, these reciprocal paths should not be interpreted as evidence of a closed or causal feedback system. The nonuniform mean-level pattern of LB across waves further underscores that wave-specific contextual influences cannot be excluded when interpreting these within-person associations.

Within the primary conservation of resources (COR) framework, the association between PE and subsequent LB can be understood as a possible resource-gain process. From this perspective, the relevance of PE may extend beyond temporarily interrupting academic tasks. In the high school context, PE may provide opportunities for physical recovery, emotional regulation, competence experiences, and peer interaction. These experiences may help students preserve or replenish the resources needed to cope with coursework, frequent examinations, and high-stakes academic evaluation. Accordingly, when the same student engaged in more PE than their usual level, greater access to these resources may have been associated with lower subsequent emotional exhaustion, academic alienation, and reduced efficacy. This interpretation is consistent with previous evidence linking PE with lower adolescent LB and with psychological resources relevant to academic adaptation (Hobfoll, 1989; Salmela-Aro and Upadyaya, 2014; Deng et al., 2025; Fu et al., 2023). However, because resource gain, coping capacity, and academic recovery were not directly measured, they should be regarded as plausible explanations rather than mechanisms demonstrated by the present data.

The association from LB to subsequent PSVU can be interpreted from the resource-loss side of the same framework. Emotional exhaustion, academic alienation, and reduced efficacy may indicate that students have fewer psychological resources available for sustained learning and effortful self-regulation. Under these conditions, short videos may become particularly attractive because they are easily accessible, require little initiation effort, and provide immediate feedback. Their use may therefore serve as a temporary form of escape-oriented regulation when students feel unable to cope effectively with academic demands, which is conceptually consistent with the reality-escape dimension of PSVU. Previous studies have similarly linked LB and academic stress with problematic forms of digital-media use and avoidance-oriented coping (Hao et al., 2022; Tomaszek and Muchacka-Cymerman, 2020; Wang et al., 2020). Nevertheless, the present study did not directly assess coping motives, emotional relief, or reward-seeking processes. These explanations therefore remain theoretically plausible interpretations of the observed longitudinal association.

The reverse association from PSVU to subsequent LB suggests that this resource-loss process may also operate in the opposite direction. Time distortion and attention conflict may interfere with learning plans and sustained academic engagement, while withdrawal discomfort may add to students’ emotional burden. Repeated disruption of learning time, attention, and recovery may consequently be associated with further depletion of learning-related resources and higher subsequent LB. Importantly, however, the RI-CLPM identified temporally ordered within-person associations rather than the intervening processes themselves. Sleep disruption, attentional depletion, time displacement, and coping motives were not directly measured, and unmeasured time-varying factors may also have contributed to the reciprocal pattern. The resource-loss interpretation should therefore be understood as a focused and parsimonious theoretical account of the findings, rather than as evidence of a demonstrated causal feedback mechanism.

Notably, the longitudinal indirect association from T1 PE to T3 PSVU through T2 LB was statistically significant but small, supporting the longitudinal indirect role of LB and H4. This finding indicates that when the same student’s PE was higher than their usual level, subsequent LB tended to be lower, and lower LB was further associated with lower subsequent PSVU severity. However, the direct PE → PSVU association was significant in the constrained M5 but showed interval-specific uncertainty in M1. LB should therefore be understood as one possible longitudinal pathway linking PE to subsequent PSVU rather than as the sole explanatory mechanism. The key value of the RI-CLPM is that this longitudinal indirect association is based on deviations from individuals’ own usual levels rather than stable differences between students. Nevertheless, the pathway may still be influenced by unmeasured time-varying factors. Accordingly, the findings support a longitudinal indirect association with temporal ordering but do not establish a causal mediation mechanism.

### 4.3 Bridging longitudinal explanation and prediction: proximal state dependence of PSVU

The machine-learning analysis was not intended to replicate the RI-CLPM findings, but rather to provide an additional layer of evidence from a predictive perspective. Whereas the RI-CLPM examined within-person longitudinal associations among PE, LB, and PSVU, the machine-learning models addressed a different question: whether these variables predicted subsequent PSVU among students who were not involved in model training. The models also tested whether PE and LB provided incremental predictive information beyond prior PSVU. Because statistical associations do not necessarily translate into out-of-sample prediction, combining explanatory longitudinal modeling with cross-validated prediction can help evaluate the generalizability of theoretically relevant variables and constrain overinterpretation (Shmueli, 2010; Yarkoni and Westfall, 2017). Compared with previous studies that mainly identified high-risk groups using concurrent digital-use characteristics, the present study used three-wave data to predict T3 PSVU, thereby further distinguishing longitudinal association from incremental predictive value (Kim et al., 2024; Lee and Kim, 2021).

The results revealed clear proximal state dependence. When only T1 information was used, the holdout test-set *R*^2^ ranged from 0.142 to 0.155. After T2 information was added, *R*^2^ increased to 0.288–0.311, with the T1 + T2 baseline XGBoost model showing the best performance (*R*^2^ = 0.311). In the extended model, T2 PSVU made the largest SHAP contribution, accounting for 45.5% of the total absolute SHAP value, followed by T2 LB, T1 PSVU, and T2 PE. This pattern indicates that problematic use states closer to the outcome wave contained more predictive information. For high school students embedded in intensive coursework, frequent examinations, and relatively fixed daily routines, once short-video use develops into time distortion, reality escape, withdrawal discomfort, and attention conflict, it may show a certain degree of behavioral continuity. Therefore, the prediction of PSVU should not be reduced to the prediction of viewing duration alone; the key issue is whether problematic patterns of use have already emerged (Marciano and Camerini, 2022).

Adding PE and LB to models that already included prior PSVU did not yield reliable improvements in holdout performance. This pattern should not be regarded as inconsistent with the RI-CLPM findings, because explanatory longitudinal modeling and out-of-sample prediction address different questions. The RI-CLPM examines whether within-person deviations in PE and LB precede subsequent changes in PSVU, whereas the predictive models assess whether these variables improve prediction once prior PSVU is already known. Proximal PSVU may summarize part of the accumulated behavioral and contextual information shared with PE and LB, thereby limiting their unique predictive contribution. Accordingly, their SHAP contributions reflect how predictive information is allocated within the fitted model, rather than independent incremental value or causal effects (Lundberg and Lee, 2017). The comparable performance of Ridge regression and XGBoost likewise suggests limited benefit from additional nonlinear complexity. Leave-one-school-out validation indicated that the predictive pattern was not dependent on same-school overlap between the development and test sets. Nevertheless, with only five schools from one regional sampling frame, this finding supports cross-school robustness within the participating sample rather than external transportability. Overall, recent PSVU appears most informative for near-term risk identification, whereas PE and LB may be more relevant to understanding the broader developmental process than to serving as independent screening indicators.

### 4.4 Implications for future school-based prevention research

Taken together, the RI-CLPM, longitudinal indirect-association, and machine-learning findings identify several candidate targets for future school-based prevention research. Recent PSVU symptoms—including time distortion, reality escape, withdrawal discomfort, and attention conflict—may warrant attention in future risk-identification research, while opportunities for PE and support for LB may be examined as complementary upstream components. However, because the study was observational, the longitudinal indirect association was small, and PE and LB did not provide stable incremental predictive value beyond prior PSVU, these findings do not establish an effective stratified intervention or demonstrate that modifying PE or LB will reduce PSVU.

The study did not measure objective short-video viewing duration and therefore cannot identify a safe-use threshold or determine the effectiveness of reducing viewing time. Future controlled or quasi-experimental studies should evaluate whether interventions addressing exercise opportunities, learning burnout, and digital self-regulation produce meaningful changes in PSVU before these approaches are recommended for routine school practice.

## 5 Limitations and future directions

First, the sample was drawn from five selected high schools in Yunnan Province, China, limiting generalizability to other regions and educational contexts. Participants were nested within five schools and 36 classrooms. Anonymous school codes enabled school-fixed-effect and leave-one-school-out RI-CLPM sensitivity analyses, which broadly supported the overall pattern but indicated some sensitivity in two comparatively small paths. However, only five schools were available, precluding reliable use of conventional school-clustered sandwich standard errors. Individual-level classroom identifiers and classroom-specific analytic sample sizes were also unavailable; therefore, classroom-level ICCs, clustered standard errors, and multilevel RI-CLPMs could not be estimated. Unmodeled within-classroom dependence may have produced underestimated standard errors and overly narrow confidence intervals, particularly for smaller effects. The primary analyses were also restricted to 1,327 participants with valid three-wave linkage and complete core-variable scores. Although baseline T1 data permitted comparison between retained and excluded participants, valid cross-wave linkage for excluded cases was not retained after de-identification. Their incomplete longitudinal records could therefore not be reconstructed as an individually linked partial dataset for FIML sensitivity analysis. Selection associated with graduation or other loss to follow-up may consequently have influenced the findings. Future studies should recruit more diverse schools and retain non-identifying school, classroom, and longitudinal linkage codes for all eligible partial records, permitting multilevel or small-cluster-robust inference and missing-data sensitivity analyses.

Second, this study used a three-wave longitudinal design with 6-month intervals. Although this design allowed the temporal associations among PE, LB, and PSVU to be examined while separating stable between-person differences from within-person fluctuations, three waves may be insufficient to characterize longer-term developmental trajectories or associations operating over shorter time scales. Future studies should incorporate additional measurement waves, longer follow-up periods, and, where appropriate, shorter assessment intervals to examine whether these associations remain consistent across developmental stages and academic periods.

Third, the study relied primarily on self-report measures, and several measurement-related limitations warrant consideration. Although longitudinal measurement invariance was supported for LB and PSVU at the parcel level, the primary RI-CLPM used observed scale scores rather than fully latent measurement models. Measurement error was therefore not explicitly separated from within-person fluctuations and may have influenced the estimated autoregressive and cross-lagged associations. In addition, parcel-level invariance may mask item-specific non-invariance and should not be interpreted as evidence that every individual item functioned identically across waves. PARS-3 represents a different measurement structure because exercise intensity, duration, and frequency form a formula-based behavioral composite rather than interchangeable reflective indicators; its longitudinal comparability was therefore based on identical administration, response options, and scoring procedures across waves rather than reflective-factor measurement invariance. Because PE, LB, and PSVU were all assessed by self-report from the same participants, the observed associations may also remain susceptible to recall bias, social desirability, shared-method variance, and occasion-specific measurement error. Harman’s single-factor test provides only a limited diagnostic of whether a single factor dominates the covariance structure and cannot establish the absence or practical insignificance of common method bias. Importantly, although the three-wave longitudinal design provides temporal ordering and the RI-CLPM separates stable between-person differences from within-person fluctuations, neither approach eliminates shared-method variance or occasion-specific measurement error. Future studies should consider item-level longitudinal measurement models and multiple-indicator latent RI-CLPMs that explicitly account for measurement error, together with objective or device-assisted measures of exercise intensity, duration, and frequency, digital-use records, and multi-informant assessments.

Fourth, although the RI-CLPM separates stable between-person differences from within-person temporal fluctuations, it does not eliminate unmeasured time-varying confounding. Examination pressure, short-term academic stress, sleep, school schedules, peer relationships, and changes in digital-media exposure may vary across waves and simultaneously influence PE, LB, and PSVU. This issue is particularly relevant because assessments were conducted in March 2025, September 2025, and March 2026, and LB showed a nonuniform descriptive pattern across these waves, increasing from 47.67 at T1 to 50.24 at T2 before decreasing to 47.36 at T3. Because these time-varying contextual factors were not repeatedly measured, their contribution to the observed cross-lagged and longitudinal indirect associations cannot be excluded. Accordingly, these estimates should be interpreted as within-person longitudinal associations after accounting for stable between-person differences, rather than as causal effects or causal mediation. Future studies should repeatedly assess major time-varying academic, behavioral, and contextual factors and incorporate them as time-varying covariates; where feasible, experimental or quasi-experimental designs would provide stronger evidence regarding causal processes.

Fifth, the present study focused on the core longitudinal associations among PE, LB, and PSVU and therefore did not capture the broader multilevel context in which adolescent digital behavior develops. Individual, family, peer, school, and platform-level factors may jointly shape these processes, and their effects may vary across developmental and educational contexts. Future research could integrate variables such as sleep quality, peer relationships, family environment, school climate, examination periods, and platform algorithmic exposure to develop a more comprehensive account of the factors associated with PSVU.

Finally, the machine-learning analyses should be regarded as supplementary and exploratory rather than as causal validation of the RI-CLPM findings. The original development and holdout sets were divided at the student level, allowing students from the same schools to appear in both datasets; these results therefore represent internal prediction in new students from the same source population. The additional leave-one-school-out analysis provided a more stringent assessment and showed no marked deterioration in predictive performance. Nevertheless, because it included only five schools from the same regional sampling frame, it should be interpreted as a school-grouped sensitivity analysis rather than definitive external validation. Classroom-grouped validation could not be conducted because individual classroom identifiers were unavailable. Although the extended models yielded higher point estimates under leave-one-school-out validation, five school folds were insufficient for reliable inferential comparisons and did not establish stable incremental predictive value for PE and LB beyond prior PSVU. The current models should not be used for individual screening or school-level decision-making, and SHAP values should be interpreted only as model-specific attributions within the extended model, not as evidence of causal effects or incremental predictive value beyond the baseline model. Future studies should use larger numbers of schools, classroom-grouped resampling, independent external samples, and richer behavioral and contextual predictors.

## 6 Conclusion

This study identified stable between-person associations and an overall pattern of temporally ordered within-person associations among PE, LB, and PSVU in Chinese high school students. The constrained M5 indicated that higher-than-usual PE was associated with lower subsequent LB and PSVU, whereas higher-than-usual LB and PSVU were associated with lower subsequent PE. The unconstrained M1 retained the same path directions but showed interval-specific uncertainty in the comparatively weak PE → PSVU and LB → PE associations. By contrast, PE → LB, the reciprocal associations between LB and PSVU, and PSVU → PE were supported across both unconstrained intervals. T1 PE was also weakly associated with T3 PSVU through T2 LB, suggesting that LB may represent one longitudinal pathway linking PE to later PSVU.

The machine-learning analysis further showed that recent PSVU was the most informative predictor of subsequent PSVU, whereas PE and LB did not provide clear incremental predictive improvement beyond prior PSVU. This distinction underscores that longitudinal association modeling and out-of-sample prediction address different questions. Overall, the findings may help identify candidate targets for future school-based intervention research, but they do not demonstrate that changing PE or LB will reduce PSVU. Multisource measurement, independent-sample validation, and experimental or quasi-experimental intervention studies are needed to determine whether these associations can be translated into effective prevention approaches.

## Data Availability

The datasets presented in this article are not readily available because they contain sensitive information from adolescent participants, and public sharing is restricted by the conditions of the ethics approval and informed consent. De-identified data may be made available by the corresponding author upon reasonable request and subject to applicable ethical approval. Requests to access the datasets should be directed to Zeng Gao, wwzgaozeng@163.com.
