# Supplementary material for: Does learning burnout link physical exercise and problematic short-video use? Evidence from a three-wave random-intercept cross-lagged panel model and supplementary machine-learning analysis among Chinese high school students
Source: Front Psychol. 2026 Oct 1;17:1929181. doi: 10.3389/fpsyg.2026.1929181 (PMC13668801; doi:10.3389/fpsyg.2026.1929181)
Supplement: Supplementary file 1 [file Table_1.docx]

Supplementary Material

Table S1. STDYX-standardized estimates from the unconstrained RI-CLPM (M1)

Panel A. Between-person associations

| Path | *β* | *SE* | *p* |
| --- | --- | --- | --- |
| RI-PE ↔ RI-LB | −0.467 | 0.126 | <0.001 |
| RI-PE ↔ RI-PSVU | −0.268 | 0.078 | 0.001 |
| RI-LB ↔ RI-PSVU | 0.444 | 0.143 | 0.002 |

Panel B. Autoregressive and cross-lagged effects

| Effect | Path | T1 → T2 | | | T2 → T3 | | |
| --- | --- | --- | --- | --- | --- | --- | --- |
|  |  | *β* | *SE* | *p* | *β* | *SE* | *p* |
| Autoregressive | PE → PE | 0.267 | 0.052 | <0.001 | 0.230 | 0.058 | <0.001 |
| Autoregressive | LB → LB | 0.368 | 0.047 | <0.001 | 0.343 | 0.047 | <0.001 |
| Autoregressive | PSVU → PSVU | 0.225 | 0.049 | <0.001 | 0.253 | 0.047 | <0.001 |
| Cross-lagged | PE → LB | −0.144 | 0.045 | 0.001 | −0.128 | 0.040 | 0.002 |
| Cross-lagged | PE → PSVU | −0.041 | 0.047 | 0.382 | −0.132 | 0.045 | 0.004 |
| Cross-lagged | LB → PE | −0.103 | 0.043 | 0.016 | −0.054 | 0.052 | 0.302 |
| Cross-lagged | LB → PSVU | 0.263 | 0.042 | <0.001 | 0.242 | 0.044 | <0.001 |
| Cross-lagged | PSVU → PE | −0.118 | 0.046 | 0.010 | −0.158 | 0.050 | 0.002 |
| Cross-lagged | PSVU → LB | 0.112 | 0.042 | 0.008 | 0.116 | 0.039 | 0.003 |

Panel C. Within-wave correlations

| Path | T1 | | | T2 residual | | | T3 residual | | |
| --- | --- | --- | --- | --- | --- | --- | --- | --- | --- |
|  | *β* | *SE* | *p* | *β* | *SE* | *p* | *β* | *SE* | *p* |
| PE ↔ LB | −0.095 | 0.059 | 0.108 | −0.056 | 0.046 | 0.218 | −0.085 | 0.036 | 0.019 |
| PE ↔ PSVU | −0.046 | 0.058 | 0.433 | −0.023 | 0.049 | 0.638 | −0.147 | 0.042 | <0.001 |
| LB ↔ PSVU | 0.159 | 0.057 | 0.006 | 0.177 | 0.041 | <0.001 | 0.093 | 0.036 | 0.010 |

*Note. β* values are STDYX-standardized estimates from M1; standardized SEs and p values are based on 5,000 bootstrap samples. PE = physical exercise; LB = learning burnout; PSVU = problematic short-video use. Demographic and educational covariates predicted the three random intercepts but are omitted for concision because they were not subject to temporal equality constraints. In M5, equality constraints were imposed on corresponding unstandardized coefficients; standardized coefficients may therefore differ slightly across intervals because they depend on wave-specific variances.
